# Update on the Management of Breast Cancer during Pregnancy

**DOI:** 10.3390/cancers12123616

**Published:** 2020-12-03

**Authors:** Francesca Poggio, Marco Tagliamento, Chiara Pirrone, Davide Soldato, Benedetta Conte, Chiara Molinelli, Maurizio Cosso, Piero Fregatti, Lucia Del Mastro, Matteo Lambertini

**Affiliations:** 1 Breast Unit, IRCCS Ospedale Policlinico San Martino, 16132 Genova, Italy; francescabenedetta.poggio@hsanmartino.it (F.P.); lucia.delmastro@hsanmartino.it (L.D.M.); 2 U.O. Oncologia Medica 2, Medical Oncology Department, IRCCS Ospedale Policlinico San Martino, 16132 Genova, Italy; tagliamento.marco@gmail.com (M.T.); chiara.pirrone.92@gmail.com (C.P.); davide.soldato@gmail.com (D.S.); bntconte@gmail.com (B.C.); chiara.molinelli91@gmail.com (C.M.); 3 Department of Internal Medicine and Medical Specialties (DiMI), School of Medicine, University of Genova, 16132 Genova, Italy; 4 Department of Radiology, IRCCS Ospedale Policlinico San Martino, 16132 Genova, Italy; maurizio.cosso@hsanmartino.it; 5 U.O.C. Clinica di Chirurgia Senologica, Department of Surgery, IRCCS Ospedale Policlinico San Martino, 16132 Genova, Italy; piero.fregatti@unige.it; 6 Department of Integrated Diagnostic Surgical Sciences, School of Medicine, University of Genova, 16132 Genova, Italy; 7 U.O.C. Clinica di Oncologia Medica, Medical Oncology Department, IRCCS Ospedale Policlinico San Martino, 16132 Genova, Italy

**Keywords:** pregnancy during breast cancer, pregnancy, breast cancer, surgery, radiotherapy, chemotherapy, targeted therapy, endocrine therapy, immunotherapy

## Abstract

**Simple Summary:**

The diagnosis of cancer during pregnancy represents a challenging situation, leading to a complex management aimed at maximizing the curative approach to the patient while minimizing potential adverse events towards the baby. The different treatment strategies can be combined according to the gestational age. However, to date, data regarding breast cancer care occurring during pregnancy are mostly derived from retrospective reports, thus the inclusion of patients in dedicated registries is advisable. This review aims at reporting the updates on this topic, giving insights into practical aspects of the management of this uncommon and demanding situation.

**Abstract:**

The diagnosis of breast cancer during pregnancy represents a challenging situation for the patient, her caregivers and physicians. Pregnancy adds complexity to oncological treatment planning, as many therapies can be potentially dangerous to the fetus. Therefore, a multidisciplinary approach is needed to offer a proper care for obtaining the best possible outcomes for the mother and the future child. Breast surgery is feasible throughout the pregnancy while radiotherapy should be postponed after delivery. Administration of chemotherapy is considered safe and can be given during the second and third trimesters, while it is contraindicated in the first trimester due to the high risk of fetal malformations. Endocrine therapy and targeted agents are not recommended during the whole pregnancy period; however, limited data are available on the use of the majority of new anticancer drugs in this context. The aim of the current review is to provide an update on the current state of art about the management of women diagnosed with breast cancer during pregnancy.

## 1. Introduction

The occurrence of cancer during pregnancy is a rare situation, with an estimated incidence of 1 case every 1000 pregnancies [1,2].

However, the number of cancers complicating pregnancy may rise in the next years, particularly due to the increased age at the first pregnancy [3]. The most common types of cancer diagnosed during pregnancy are breast cancer, cervical cancer, lymphoma, ovarian cancer, leukemia, colorectal cancer, and melanoma [4].

A diagnosis of breast cancer during pregnancy is challenging for patients, caregivers, and physicians, because the maternal benefit from treatments must be balanced with the potential harms to the fetus. Notably, this complex situation should be managed within a multidisciplinary expert team, and the proposed treatment approach should be discussed with the patient and her family, taking into account cancer prognosis, gestational age and possible risks for the fetus [5].

Considering the young age of patients with breast cancer during pregnancy, proper genetic counselling should be offered [6].

The aim of this review is to provide an update on the current state of art about the management of women diagnosed with breast cancer during pregnancy.

## 2. Breast Cancer during Pregnancy: Prognosis and Biology

Breast cancer during pregnancy has been demonstrated to be associated with a lower prevalence of hormone receptor expression, thus with a predominance of more aggressive subtypes that are peculiar for younger ages, such as triple-negative or HER2-positive. The diagnosis occurs more frequently at more advanced stages in comparison with non-pregnant patients, potentially contributing to determine a worse prognosis. This may be related to the delay in the diagnosis, to a suboptimal staging because of the teratogenicity of most radiological imaging procedures, and to the risk of suboptimal management [7,8,9]. Several studies have addressed this issue, with conflicting results, probably due to the small sample size [10,11]. A retrospective study conducted in a cohort of 75 pregnant women with breast cancer treated in the second and third trimesters with 5-fluorouracil, doxorubicin, and cyclophosphamide, compared with a cohort of non-pregnant patients matched for age, stage of disease and year of diagnosis, showed statistically significant improvement in disease-free survival (DFS), progression-free survival and overall survival (OS) for pregnant patients [11]. A further cohort study including 311 patients with breast cancer diagnosed during pregnancy (of whom the majority received systemic anticancer treatment) and 865 women with breast cancer diagnosed outside pregnancy did not find a detrimental effect of pregnancy on both DFS and OS, after adjusting for patient, tumor, and treatment characteristics [12]. The results of these studies support the concept that the diagnosis of breast cancer during pregnancy is not associated with worse outcomes per se, if patients receive standard local and systemic treatments.

To have a better understanding of the biology of breast cancer during pregnancy, Nguyen et colleagues aimed to identify specific molecular alterations of these patients, as compared to matched non-pregnant women. A genome-wide copy number alterations profiling on primary tumor samples revealed no differences between patients and controls [13]. However, a whole-genome sequencing analysis showed that pregnancy-associated breast cancer had a significantly higher number of non-silent mutations, mutations in the mucin gene family, and an enrichment of mismatch repair deficiency mutational signature and higher stromal and tumor-infiltrating lymphocyte (TILs) levels, suggesting a potential impact of pregnancy on tumor biology [13,14].

A recent systematic review has summarized the available evidence on the genomic profile of pregnancy-associated breast cancer, demonstrating the aberrant expression of several oncogenes (e.g., *MYC*, *SRC*, *FOS*), tumor suppressor genes (e.g., *TP53*, *PTEN*, *CAV1*), apoptosis regulators (e.g., *PDCD4*, *BLC2*, *BIRC5*), transcription regulators (e.g., *JUN*, *KLF1*, *SP110*), genes involved in DNA repair mechanisms (e.g., *Sig20*, *BRCA1/2*, *FEN1*), in cell proliferation (e.g., *AURKA*, *MKI67*), and in the immune response (e.g., *PD1*, *PDL1*) [15].

Further research in this area is required to shed light on the peculiar biological features of breast cancer during pregnancy.

## 3. Anticancer Treatments during Pregnancy

Guidelines recommend that breast cancer during pregnancy should be treated according to the same recommendations as breast malignancies in young non-pregnant women [7,16]. Clinicopathological characteristics, gestational age at diagnosis of breast cancer, expected date of delivery, and patients’ preferences are the crucial factors to be considered for an optimal management. A multidisciplinary approach is mandatory to obtain the best possible outcomes for the mother and the future child.

### 3.1. Local therapy

Breast cancer surgery is considered safe throughout the whole pregnancy and should follow the same recommendations as for non-pregnant women whenever feasible. Despite the frequent delayed diagnosis being linked to a larger size of breast tumor, radical surgery should not forcibly be the preferred option [7,8]. Since radiotherapy should be postponed after delivery, the type of breast surgery is mostly influenced by the expected time of radiotherapy initiation and should be discussed within a multidisciplinary team. Patients diagnosed in the first trimester, not candidates to receive chemotherapy and wishing to perform the breast conserving surgery and to pursue the pregnancy, have to be informed that a long delay in the start of adjuvant radiotherapy could result in a potential increased risk of local recurrence [17,18,19]. Despite large dedicated studies not having been conducted yet, similar survival rates were observed among women who underwent breast conserving or radical surgery [20]. To note, the majority of anesthetics can be safely used during pregnancy [21]. Immediate breast reconstruction using a tissue expander can be performed after mastectomy, without apparently increasing morbidity to the patient or the fetus. Nevertheless, physiological changes of the breast of pregnant women should be taken into account, and a delayed reconstruction procedure after delivery can be considered [17].

Sentinel lymph node biopsy in pregnant patients is a controversial topic. Recommendations from the American Society of Clinical Oncology (ASCO) do not support this procedure, while the National Comprehensive Cancer Network (NCCN) guidelines endorse this approach on the basis of several studies showing that this procedure can be safely performed [22,23]. If pregnant patients are offered sentinel node biopsy, Technetium-99m (99mTc) colloid solution injection should be the preferred option [24]. To minimize the exposure to radiation, it is preferred to adopt the one-day protocol, injecting colloid in the morning of the day of surgery [7]. Blue dye and isosulfan blue should be avoided because of its risk of inducing an allergic or anaphylactic maternal reaction, and methylene blue is contraindicated during first trimester because of its known teratogenic effect [8].

An international cohort study including 145 women with breast cancer during pregnancy reported a high identification rate and a low axillary recurrence with sentinel lymph node biopsy [25]. Hence, this approach can be considered safe in clinically node negative pregnant patients with the use of radioactive colloid and the one-day protocol [7].

### 3.2. Chemotherapy

The administration of chemotherapy in women with breast cancer during pregnancy should be done following the guidelines for non-pregnant patients as closely as possible [7,16]. Risks for the fetus vary according to gestational age; therefore, the indication for chemotherapy in this setting strongly depends on the timing of breast cancer diagnosis.

Before starting any oncological treatment, a fetal examination with ultrasound should be performed to exclude pre-existing abnormalities [8].

During the first trimester, the exposure to cytotoxic agents can interfere with fetal organogenesis, resulting in an increased risk of miscarriages and in congenital malformations in about 14% of cases [26,27,28]. Therefore, chemotherapy is contraindicated during the first trimester [7,26,27,28]. If there is an urgent need to start chemotherapy in this timespan, the option of terminating the pregnancy in order to avoid the delay in treatment initiation should be carefully discussed with patients [9,29].

After the first trimester, the prevalence of fetal malformations due to chemotherapy regimens drops to 3%, similarly to what observed in the general population. Thus, chemotherapy can be safely administered in the second and third trimesters, always with a close surveillance of the mother and the fetus [7,16]. The blood placenta barrier limits the fetal exposure to cytotoxic drugs, with a different transplacental capacity for different drugs [30,31].

Currently, anthracyclines, cyclophosphamide, and taxane-based regimens represent the standard of care for the (neo)adjuvant treatment of breast cancer patients [6,32,33]. Platinum compounds may have a role in the neoadjuvant treatment of triple negative breast cancer patients [32,34].

Table 1 summarizes the main results on the use of anthracyclines and taxanes during pregnancy in terms of pregnancy complications and fetal outcomes [35,36,37,38,39,40,41,42,43]. Anthracyclines and alkylating agents such as cyclophosphamide have been known to be safe in pregnant women for many years; recently, more reassuring evidence on the safety use of taxanes during pregnancy has become available (see Table 1). Potential fetal toxic effects of platinum-derivatives are scarce due to their less frequent implication in standard treatment protocols for breast cancer [4]. One study reported platinum-based chemotherapy to be associated with smaller size for gestational age [4]. Overall, regimens containing anthracyclines and taxanes appear to be safe in the second and third trimester of pregnancy [44].

The use of dose-dense chemotherapy (by reducing the intervals between treatment cycles and intensifying the administered dose) has been considered a standard of care for the treatment of high risk breast cancer patients [45,46]. A pooled analysis conducted specifically in premenopausal patients showed a significant improvement in terms of DFS and OS with the use of dose-dense chemotherapy in this setting [47]. Despite dose-dense being the preferred option for premenopausal patients with a higher risk of relapse, few data are available with the use of this schedule in patients with breast cancer during pregnancy. A retrospective cohort study conducted in 109 pregnant women with breast cancer, of whom 10 received dose-dense chemotherapy, showed the safety of this approach [48]. However, the small sample size limits the possibility to counsel patients on the safety of dose-dense chemotherapy during pregnancy.

As in non-pregnant women, chemotherapy dosing should be based on the current body surface area [7,8,16]. The physiological changes occurring during pregnancy (i.e., increase in plasma volume, increase in glomerular filtration rate, changes in albumin concentration) may result in lower maximal concentration and decreased exposure to chemotherapy [3,49]. Therefore, underdosing should be avoided in pregnant women. On the other hand, whether chemotherapy doses have to be increased is uncertain, as overdosing may result in fetal toxicity.

After week 35 of gestation, chemotherapy should be stopped to allow a 2/3-week interval prior to delivery to prevent hematological complications during the delivery [3,50].

### 3.3. Endocrine Therapy

Endocrine therapy is contraindicated for the treatment of breast cancer during pregnancy. In animal models, tamoxifen produces teratogenic effects, involving more frequently the genitourinary tract, and increases the risk of developing breast cancer in the offspring [51,52]. A systematic review summarized the data of 248 pregnancies in breast cancer patients exposed to tamoxifen during pregnancy [53]. Among 68 at term pregnancies, 12 babies (18%) were born with major malformations (including ambiguous genitalia, Pierre Robin sequence, and oculoauriculovertebral dysplasia), 2 babies (3%) with minor malformations (preauricular skin tags and severe hypermetropia) and 54 (79%) without any malformation. The rate of major malformations after tamoxifen exposure was 17.6%, while in the general non-exposed population it was around 3%; however, some of the babies with major malformations were also exposed to multiple teratogenic agents (e.g., other antineoplastic drugs, X-ray and other potentially teratogenic drugs). Finally, tamoxifen elicits cellular alteration similar to diethylstilbestrol, being associated with long-term adverse events becoming more evident later in life; therefore the impact of tamoxifen exposure may not be only limited to major malformations observed at birth [54]. Given the absence of data on long-term pediatric outcomes, the increased rate of major malformations in animal and human pregnancies and some confounding factors in the available data, no definitive conclusion on the teratogenic impact of tamoxifen exposure can be drawn. International guidelines contraindicate the use of tamoxifen during pregnancy and, in case of accidental exposure, drug administration should be promptly stopped if the patient desires to continue the pregnancy [16]. In patients wishing to conceive, a 3-month wash-out period is recommended after stopping tamoxifen [55]. No data are available for aromatase inhibitors exposure during pregnancy, even though their teratogenic potential has been studied in animal models [56].

### 3.4. Targeted Therapies

Targeted therapies for the treatment of breast cancer have been increasingly used in the last few years [57].

The monoclonal antibody anti-HER2 trastuzumab is the standard of care for HER2-positive breast cancer. Current guidelines contraindicate the use of trastuzumab during pregnancy, mainly due to the increased risk of developing oligo- and/or anhydramnios, as well as due to unknown long-term consequences on the fetus [7]. A systematic review and meta-analysis of 17 studies was conducted to evaluate the safety of trastuzumab during pregnancy [58]. A total of 18 pregnancies and 19 newborns were described. Trastuzumab was given in the first trimester in 16.7% of patients and in the second/third trimester in 80% of the cases. The most frequent adverse event was the occurrence of oligo/anhydramnios; interestingly, its incidence varied significantly according to the time of exposure, occurring in 73.3% of the fetus exposed during the second/third trimester as compared to none of those exposed in the first trimester.

Of note, after a median follow-up of 9 months, all children exposed to trastuzumab only in the first trimester were healthy without evidence of congenital malformations. These results are discordant with those obtained with chemotherapy, where a 20% risk of congenital malformations were reported when it was administered in the first trimester. The oligo/anhydramnios associated with the use of trastuzumab may be related to the inhibition mediated by trastuzumab of the epithelial growth factor receptors (EGFR) expressed on fetal kidney, resulting in decreased production of amniotic fluid. This eventuality happened after month 4 of gestation, contextually to the development of fetal kidney, explaining the increased risk of oligo/anhydramnios in the second and third trimester [59]. Moreover, trastuzumab is a large molecular requiring active trans-placental barrier transport through a specific mechanism that is not active during the early phase of gestation [60]. Notably, the production of amniotic fluid restarts after trastuzumab interruption, since this effect appears to be reversible, and no fetal cardiac toxicities were reported in the above study. While some physicians support counseling patients to prematurely interrupt the pregnancy in case of accidental exposure to trastuzumab during the first trimester [61], no data on major malformations have been reported so far. Therefore, although trastuzumab use during pregnancy remains contraindicated, a short accidental fetal exposure in the first trimester should not require pregnancy termination. These uncertainties should be discussed with patients, and a close monitoring in the case of pregnancy continuation should be considered.

Lapatinib is an anti-HER2 tyrosine kinase inhibitor approved for the treatment of patients with HER2-positive metastatic breast cancer [62]. Being a small molecule, it is expected to be able to cross the placenta during all phases of pregnancy. One case report described no fetal malformations nor pregnancy complications in a women who accidentally became pregnant while on treatment with lapatinib for metastatic breast cancer and stopped it in the first trimester [63]. A recent analysis of 2 randomized phase III trials testing lapatinib in the (neo)adjuvant setting reported on the pregnancies that occurred during or after treatment. Despite both protocols requiring active contraception for one year, 12 women exposed to trastuzumab and/or lapatinib became unintentionally pregnant during anti-HER2 therapy or soon after its completion: 7 patients (58.3%) opted for an induced abortion, and 5 (41.7%) completed the pregnancy (2 were exposed to trastuzumab within the 7 months since their last menstrual cycle, 1 had active unintentional short-term exposure to trastuzumab, 1 to lapatinib only and 1 to both). All pregnancies and deliveries had no complications, resulting in live births without congenital anomalies [64].

In the last few years, several anti-HER2 agents, especially pertuzumab, trastuzumab-emtansine (T-DM1) and neratinib, are being used in the early setting. Up to now, no data are available on their administration in pregnant women, thus they are contraindicated.

Results of the ongoing prospective US registry MotHER, collecting information on patients exposed during pregnancy or within 7 months prior to conception to trastuzumab, pertuzumab or T-DM1, will define how to properly counsel patients on this issue (ClinicalTrials.gov: NCT00833963), although currently these agents are contraindicated during pregnancy [7].

In luminal disease, recently, the addition of cycline-dependent kinase 4/6 inhibitors (CDK4/6i) abemaciclib to endocrine therapy showed promising results in the adjuvant setting for patients at a high risk of relapse leading to a potential role of these drugs in the early stage in next few years [65]. To date, no data on the safety of CDK4/6i during pregnancy are available, so currently their use is contraindicated during pregnancy.

### 3.5. Supportive Care

Chemotherapy-induced nausea and vomiting (CINV), as well as allergic reactions, are the most common side effects of chemotherapy. Most of the preventive drugs normally used for non-pregnant patients can be safely administered also during pregnancy. Ondansetron is the most studied drug in this class and can be safely administered [66,67,68]. Two recent metanalyses confirmed the absence of increased risk of major malformations after ondansetron administration during the first trimester [69,70]. The histamine type 2 receptor (H2) antagonist, mostly used for premedication of taxanes, can be used to prevent allergic reactions without an increased risk of major malformations [71].

Corticosteroid administration is commonly used to prevent CINV and anaphylactic reactions. In an animal model, the administration of dexamethasone during the first trimester of pregnancy has been associated with an increased risk of major malformations including cleft palate, impaired kidney function, low-birth weight and impaired brain development [72,73]. Evidence from human studies is less compelling: retrospective studies suggested a significant impact of dexamethasone administration on cognitive and metabolic outcomes, even though prospective studies with a longer follow-up are required to further elucidate these aspects [74,75]. Given the possible adverse outcomes associated with dexamethasone, methylprednisolone should be the steroid of choice in pregnant patients [76].

No published evidence is available regarding the safety of Neurokinin1 (NK1) inhibitors during pregnancy.

Granulocyte-Colony Stimulating Factors (G-CSFs) are widely used for the prevention and treatment of febrile neutropenia, especially in dose-dense schedules. Limited data have been produced on the use of G-CSF in pregnant women. One retrospective cohort study assessing the use of G-CSF during chemotherapy in pregnant women found no differences in terms of gestational age at birth, incidence of congenital anomalies and neutropenia in exposed babies [77]. Furthermore, data on pregnant women treated with G-CSF for severe chronic neutropenia showed no increased risk of fetal death or congenital anomalies [78]. In conclusion, if a dose-dense chemotherapy regimen is deemed necessary, treatment with G-CSF should not be withheld.

### 3.6. Immunotherapy

Immunotherapy with antibodies directed against programmed cell death protein 1 (PD-1) or its ligand (PD-L1) is becoming a relevant option in the treatment of breast cancer, especially for triple-negative subtype [79,80,81]. During pregnancy, the mother develops an immune tolerance towards the fetus, involving the PD-1/PD-L1 pathway; therefore, its inhibition could potentially result in an immune response against the fetus [82]. Data derived from the use of anti-PD-1/PD-L1 in pregnant animal models reported an increased rate of third trimester miscarriages, premature delivery and birth mortality [83].

Immunotherapy during pregnancy is contraindicated until additional data on the safety of these compounds in this setting become available.

**Figure 1:**
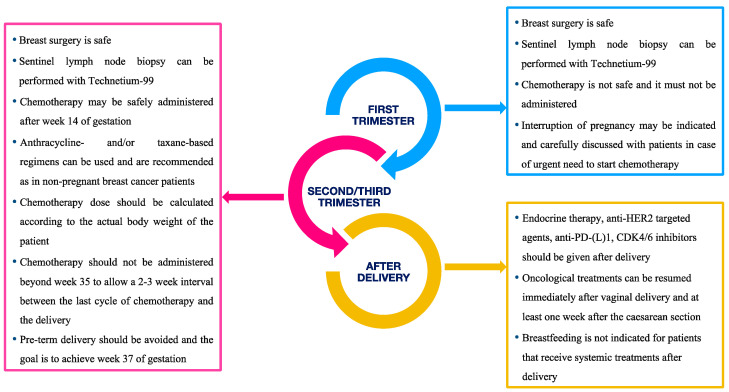
Summary of management’s recommendations according to gestational age or post-partum period.

Figure 1 reports the take home messages about the management of breast cancer during pregnancy according to the trimester of diagnosis as well as immediately after the delivery.

## 4. Impact of Breast Cancer Treatment on Pregnancy Outcomes

An important concern when treating breast cancer during pregnancy is represented by a potential negative impact of anticancer treatments on fetal health.

Although chemotherapy can be administered during the second and third trimesters without increasing the risk of malformations, a higher risk of pregnancy complications cannot be excluded.

A retrospective population study reported an increased risk of stillbirths, small gestational age and preterm delivery in exposed births (defined as maternal cancer diagnosed during pregnancy or within 1 year after pregnancy) as compared to unexposed births. Among 984 cases of breast cancer during pregnancy, an increased risk of stillbirth assessed as small gestational age, preterm delivery, and neonatal mortality largely caused by iatrogenic preterm birth was reported; thus, careful monitoring on fetal growth and decisions on the timing of the delivery, aiming to a full term delivery, are critical to reduce neonatal morbidity and mortality [84].

A population-based cohort study including 11,846,300 births was specifically conducted to examine the effect of breast cancer during pregnancy on fetal/newborn outcomes. Among these patients, an increased risk of prematurity (Odds Ratio—OR −4.84, 95% CI 4.05–5.79) and rupture of membranes (OR 1.79, 95% CI 1.06–3.05) was reported, as compared to pregnant women without cancer. No associations were instead observed with intrauterine growth restriction or congenital anomalies [85], and no information on the specific systemic treatment were available to draw conclusions on the potential correlation with premature births.

A recent study aimed to explain the increased gestational complications, hypothesizing a direct damage of anthracycline on the vascularization of placenta. After administration of doxorubicin to pregnant mice during the second trimester, the authors observed a vascular-derived placental toxicity, with a reduced blood flow, and a lower birth weight. Furthermore, a histological section of the placenta of women exposed to chemotherapy revealed a decreased neovascularization and higher proliferation and apoptosis as compared with gestational age-matched chemo-naïve women [31].

Recently, a descriptive cohort study included 1170 pregnant patients with cancer (39% with a diagnosis of breast cancer), aimed to analyze changes over time in oncological, obstetrical, and neonatal outcomes. The authors observed an increased use of chemotherapy during pregnancy, in parallel with an increase of live births, and a reduction in iatrogenic pre-term deliveries, encouraging the management of these patients in obstetric high-care units [4].

After diagnosis and before administration of anticancer treatments, an accurate fetal evaluation should be done to exclude pre-existent malformations [68]. Then, an ultrasound evaluation of the fetus and of the amniotic fluid should be done at least every three weeks during treatment, mainly to exclude fetal growth restriction [7]. Regarding the mother’s health, an assessment of blood pressure and proteinuria should be done before each cycle [9]. The mode of delivery should not differ from indications in women without cancer.

The main data on the long-term complications of in utero exposure to chemotherapy almost derived from retrospective cohort studies, with some exceptions, including several different types of malignancies (see Table 2) [41,43,86,87,88]. The results of these studies are reassuring, reporting that cognitive, psychological, neurological development, as well as the cardiac outcome, of children after intrauterine exposure to chemotherapy are similar to those of babies born from healthy women. Particularly, a large prospective case-control study conducted by Amant and colleagues evaluated 129 children whose mothers received anticancer treatments during pregnancy (all cycles of chemotherapy were administered after the first trimester) that were compared with 129 matched children born from women without cancer. The study reported that, despite the incidence of preterm birth and small gestational age being higher among the exposed group, the development was normal, and chemotherapy had no clear adverse effects on growth, cognitive, and cardiac function in early childhood, suggesting that the diagnosis of cancer in pregnancy should not be an indication of pregnancy interruption. The only factor associated with a worse cognitive outcome was prematurity, irrespective of anticancer treatments [87]. Then, mothers may be aware that antineoplastic treatment after the first trimester may not be considered detrimental for the fetus, and, although there is a higher likelihood of prematurity as compared with the general population, the risk of developing complications does not differ between babies born from women without cancer during pregnancy.

Therefore, a full-term delivery is strongly recommended, as well as the best-tailored management strategy to optimize the obstetric and neonatal outcomes.

Larger prospective data collection in dedicated registries of short- and long-term complications after intrauterine exposure to chemotherapy are warranted to help oncologists in the management of patients diagnosed during pregnancy.

## 5. Conclusions

Breast cancer during pregnancy is a challenging and delicate situation requiring a multidisciplinary team work to establish the best strategy for assuring safe care for both the mother and the fetus.

**Figure 2:**
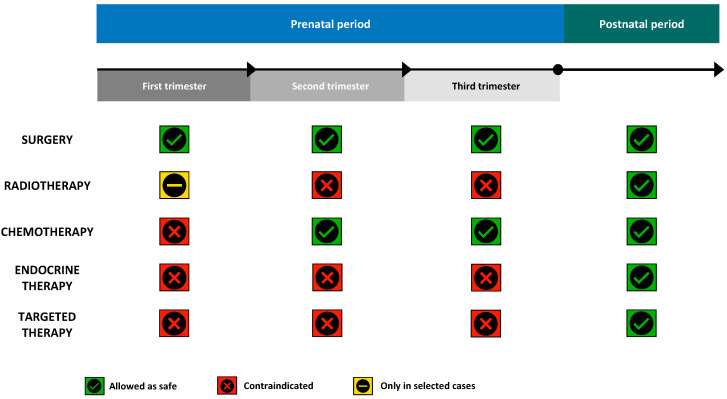
Overview of the use of anticancer treatments for breast cancer diagnosed during pregnancy.

Figure 2 gives an overview of the therapeutic options for breast cancer diagnosed during pregnancy, summarizing the indications and contraindications according to the gestational age.

Patients diagnosed with breast cancer during pregnancy can be safely treated with chemotherapy starting from the second trimester. The choice of the regimen should follow the guidelines for non-pregnant patients, anthracyclines and taxanes being the standard of care after the first trimester. Endocrine therapy and targeted therapies are not indicated in pregnant patients and should be postponed after delivery. Patients with breast cancer during pregnancy should undergo a close fetal monitoring, and a full-term delivery should be reached to reduce the risk of long-term complications.

The treatment landscape of breast cancer is rapidly evolving, but very few data have been reported about the safety of new compounds during pregnancy. The collection of prospective data regarding patients with breast cancer during pregnancy into dedicated registries is highly recommended, in order to enrich current knowledge on this topic and to improve the counseling of patients and their caregivers.

## Figures and Tables

**Table 1:** Main results of the studies assessing the use of anthracyclines and taxanes-based regimens in patients with breast cancer during pregnancy (with at least 10 patients evaluated).

Study, Year	Patients Treated with Chemotherapy, N	Type of Regimen	Gestational Age at the Beginning of CT, Weeks	Gestational Age at Delivery, Weeks	Pregnancy Complications and Fetal Outcomes
Giacalone, 1996 [35]	29	FEC, FAC, EC, VEM, VA, FA	26	34.7	35% obstetrical complications 10% spontaneous abortion 5% stillbirth No malformations
Ring, 2005 [36]	28	AC, EC	20	37	19% obstetrical complications 4% spontaneous abortion No stillbirths 4% malformations
Peccatori, 2009 [37]	20	Weekly epirubicin	Not reported	35	10% obstetrical complications No abortion No stillbirths 5% malformations
Garcia-Manero, 2009 [38]	15	A and Docetaxel, FAC	Not reported	Not reported	27% obstetrical complications No spontaneous abortion, stillbirths and malformations
Cardonick, 2010 [39]	104	AC, FAC, EC, FEC, anthracycline followed by taxane	20.4	35.8	24% obstetrical complications 5% spontaneous abortion 4% malformations Stillbirths not reported
Loibl, 2012 [40]	197	A, E, AC, EC, FAC, FEC, anthracycline followed by CMF. Anthracycline followed by taxane	24	37	17% obstetrical complications 4% malformations 1% spontaneous abortion and stillbirth
Murthy, 2014 [41]	81	FAC	Not reported	37	33% obstetrical complications 4% malformations Spontaneous abortion and stillbirths not reported
Safi, 2019 [42]	18	A, E, Docetaxel, Paclitaxel,	20 weeks	35.7	12% obstetrical complications No stillbirths and congenital malformations
O’Laughlin, 2019 [43]	50	AC plus taxane	Not reported	Not reported	Obstetrical complications were not significantly different as compared to patients treated with only anthracycline

Abbreviations A: Doxorubicin; AC: Doxorubicin, cyclophosphamide; CMF: Cyclophosphamide, methotrexate, 5-fluorouracil; CT: Chemotherapy; E: Epirubicin; EC: Epirubicin, cyclophosphamide; FA: 5-fluorouracil, doxorubicin; FAC: 5-fluorouracil, doxorubicin, cyclophosphamide; FEC: 5-fluorouracil, epirubicin, cyclophosphamide; VA: Vincristine, doxorubicin; VEM: Vincristine, epirubicin, methotrexate.

**Table 2:** Main results of the studies reporting short- and long-term outcomes following in utero exposure to chemotherapy.

Study, Year	Type of Mother’s Cancer	Type of Systemic Treatments	Results
Hahn, 2006 [86]	Breast cancer	5′-fluorouracil, doxorubicin and cyclophosphamide	No stillbirths, miscarriages, or perinatal deaths 1 subarachnoid hemorrhage, 1 Down’s syndrome
Murthy, 2014 [41]	Breast cancer	5′-fluorouracil, doxorubicin and cyclophosphamide	No significant impairment of baby health at delivery and up to childhood 3 babies with congenital abnormalities
Amant, 2015 [87]	Breast cancer, hematological malignancies, cervical cancer, ovarian cancer, brain tumor, colon cancer, gastric cancer, renal cell cancer, tongue cancer, lung cancer, thyroid cancer, melanoma, sarcoma	Anthracycline-based, anthracycline plus taxane, CMF, taxane-based, platinum-agents, vinca alkaloid, dacarbazine, temozolomide, mitoxantrone	Comparison with control group (babies from healthy women without in utero exposure to chemotherapy): - Normal cognitive, cardiac and general development - Prematurity was correlated with worse cognitive outcome, irrespective of cancer treatment
Cardonick, 2015 [88]	Breast cancer, ovarian cancer, hematological malignancies	Anthracycline-based, anthracycline plus taxane, taxane-based	Comparison with control group (babies from women also diagnosed with a cancer during pregnancy, but without in utero exposure to chemotherapy): - Higher incidence of premature birth - No significant differences in cognitive development and behavioral competence
O’Laughlin, 2019 [43]	Breast cancer	Anthracycline plus taxane	Comparison with taxanes administration delayed to the postpartum period: - Normal neonatal development - No medical disorders

Abbreviations: CMF: Cyclophosphamide, methotrexate, 5′ fluorouracil.
